# Kitchen garden, dietary diversity, and women’s health in rural eastern India: a mixed method study

**DOI:** 10.3389/fpubh.2025.1465169

**Published:** 2025-02-19

**Authors:** Shrobana Hazra, Swayam Pragyan Parida, Prajna Paramita Giri, Binod Kumar Behera

**Affiliations:** All India Institute of Medical Sciences, Bhubaneswar, India

**Keywords:** kitchen garden, dietary diversity score, women of reproductive age group, nutrition, food security

## Abstract

**Background:**

Nutrition and food security have been a development priority for decades and remain a major challenge for developing nations like India. Although agriculture is the dominant occupation in India, the rural populations experience poor nutritional outcomes and lag in socio-economic progress. More than half of the Indian women (15–49 years) are anaemic & one-third of the children are stunted. Nutrition-sensitive agricultural practices such as kitchen garden have proven as sustainable methods for reducing undernutrition at affordable costs in different regions. This study aimed to see the relationship between kitchen garden and dietary diversity by using DDS (Dietary Diversity Score) scale in Odisha, India.

**Objectives:**

To assess the relationship between dietary diversity score (DDS) and backyard kitchen garden & other socio-economic (SE) factors and to explore the enablers & barriers associated with developing a kitchen garden (KG) in rural households of Odisha, India.

**Methods:**

The study used simple random sampling to select 150 participants (WRA group: women of reproductive age) from the eligible household lists. The outcome variable for the investigation was DDS, whereas KG and SE indicators served as predictors/exposure variables. Furthermore, the study used purposive sampling to choose members for FGDs (Focus Group Discussions) to explore enablers and constraints related to growing a KG.

**Results:**

Women who did not have a household kitchen garden, had poor dietary outcomes, with DDS <5 (OR: 0.163, *p* = 0.001). Furthermore, a lack of agricultural land lowered DDS (OR: 0.176, *p* = 0.008) as well. The remaining SE parameters did not demonstrate a statistically significant relationship with DDS/diet quality. The enablers and constraints to building a KG were synthesised from 2 FGDs & further classified into four themes: seasonal fluctuation, local government’s initiatives, men’s engagement, and challenges.

**Conclusion:**

Kitchen garden can improve DDS and nutritional outcomes for the WRA group in rural Odisha. However, the distribution of seeds/saplings and small financial assistance from the local government can help with sustainability, particularly in the lower SE strata.

## Introduction

Nutrition and food security have been a development priority for decades worldwide and remain a major challenge for developing nations like India. In the Global Hunger Index-2023, India ranks 111th out of 121 qualifying countries with a score of 28.7 (1). Evidence also suggests that two-thirds of India’s current workforce is undernourished, and because of the enormous economic costs incurred it has reduced the country’s future per capita income (2, 3). Furthermore, undernutrition also accounts for 20% of maternal deaths either directly or indirectly (4). In India, undernutrition is more prevalent in rural than urban areas presumably due to poor socio-economic conditions, lack of resources, religious taboos, limited education & awareness etc. (3, 5, 6). Although agriculture is the dominant occupation in rural India, dietary quality of the rural populations remains poor which includes cheap, starchy foods and limited consumption of micro-nutrient-rich foods (fruits, vegetables and animal protein) (7). There is no exception in Odisha, an eastern Indian state. The prevalence of various nutritional deficiencies is quite high in the state (5). For example, 32% of children aged 0 to 5 are stunted and 66% of WRA (non-pregnant) are anaemic (5). Furthermore, 30% of men (15–49 years) are also anaemic in the state (5). The data presented above highlights the severity of the undernutrition issue in Odisha, which has persisted for many generations. The state is also battling poverty and a low female literacy rate (67% in rural) (5, 8) which are two predominant social determinants of undernutrition among women & children. A study conducted in Namibia and Ghana found that women of middle- or high-income households had better nutritional outcomes (4). Another study from rural West Bengal, India reiterates the similar conclusion that undernutrition is adversely correlated with factors such as the mother’s education, father’s occupation, economic status, and sanitation (9). Educational status plays an important role, possibly due to better awareness.

Different studies from around the globe have suggested tailored approaches to combat undernutrition which include nutrition-specific & nutrition-sensitive interventions (10). Nutrition-specific interventions include nutritional supplements and behavior change communication and on the /other hand, nutrition-sensitive interventions include sustainable agricultural practices at affordable cost for the community (10). Most of the Indian states have adopted interventions related to supplementary nutrition programs and awareness campaigns (11) which are mostly nutrition-specific interventions (12). However, notably, the state of Odisha had initiated an additional nutrition-sensitive intervention: ‘kitchen garden’ which primarily aimed to provide a diversified menu to the household along with reducing expenses for food and preventing those vulnerable families from falling further into the poverty trap (13). The role of small scale agriculture like kitchen garden in reducing undernutrition is undisputable across the globe (14). Furthermore, small-scale fruit and vegetable production via kitchen garden projects were identified as nutrition-sensitive agriculture interventions having the highest success rate due to their ease of adaptability (14). Another study from Melghat, India showed positive association between dietary diversity & kitchen garden and reiterated it as a sustained method for reducing malnutrition (15). Similarly, a study from rural Rwanda, Africa concluded that kitchen garden along with nutrition education can bring remarkable increase in DDS (16). The kitchen garden initiative by the local government in Odisha, included the provision of financial support & capacity building support to vulnerable households for growing their backyard kitchen garden (13).

**Figure 1:**
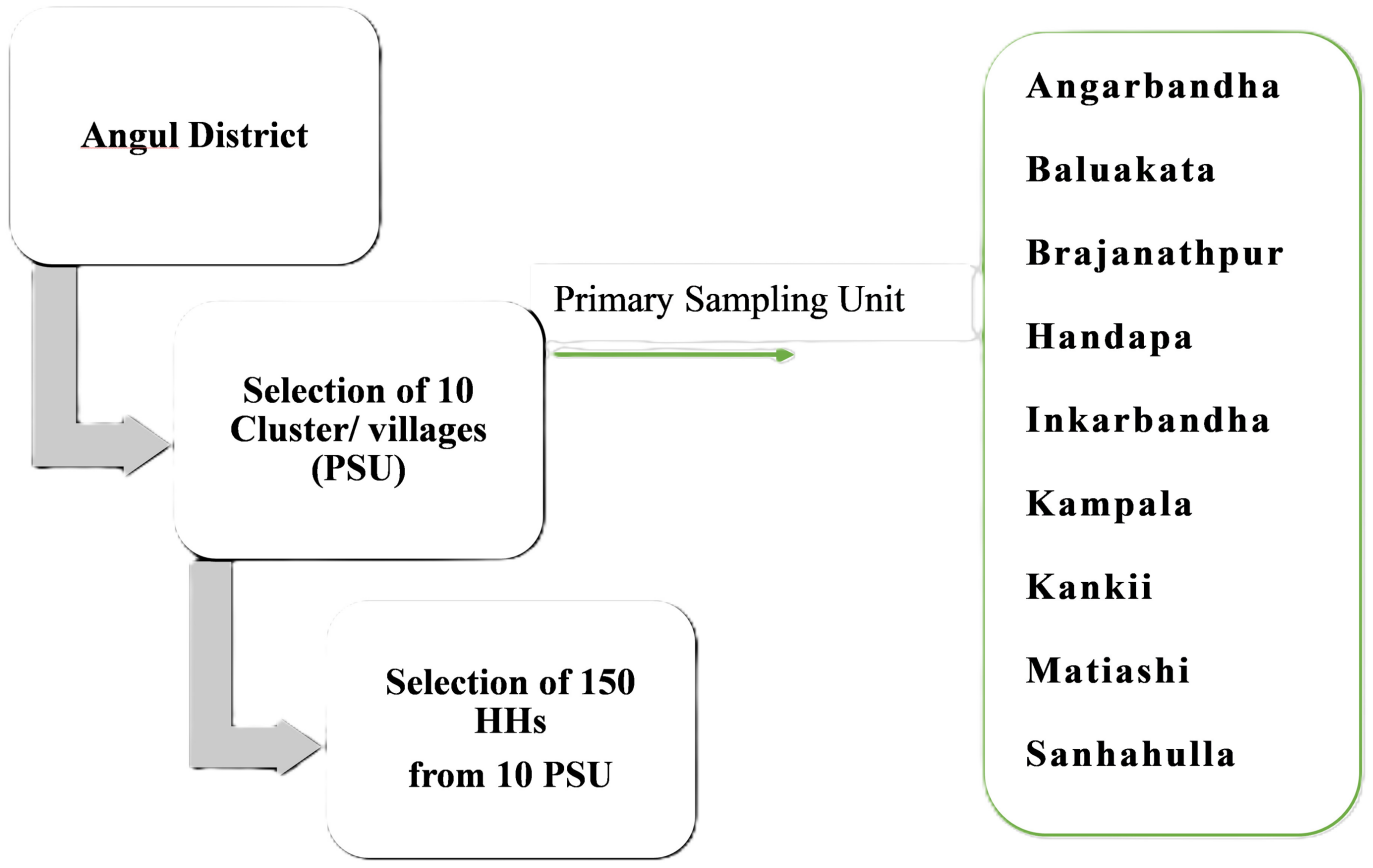
Sampling technique for quantitative data collection.

Although several studies published in the last 15 years have focused on agriculture interventions and their impact on nutrition-related outcomes, the states of India primarily focused on awareness generation programs rather than leveraging the huge agriculture resources to combat undernutrition in rural areas. Secondly, only a handful of studies were found in rural India which strengthens the case for continued research. Our study intended to examine the relationship between kitchen garden and DDS among the WRA of rural Odisha. The previous studies suggested using DDS as a proxy indicator in resource-poor settings for measuring undernutrition since it could be used without any tools or trained professionals, making it feasible to use in rural India (17). Thus, this study aimed to address the empirical question of the “relationship between kitchen garden & dietary diversity score and does it vary across the different socio-economic factors”. Furthermore, it is also critical to comprehend the viability of kitchen garden in rural areas among low socio-economic strata. However, there is almost non-existent literature in India to assess the feasibility & sustainability of kitchen garden. This study intended to examine the different enablers and barrier factors for growing and maintaining a backyard kitchen garden in rural Odisha (see Figure 1).

## Methodology

### Study settings

The study employed original data from Odisha’s Angul district. We had planned to select 2–3 additional districts from various sections of the state, but due to logistical constraints, only one district could be picked on purpose. Since the data collection took place in January–February 2022, soon after India’s second wave of COVID-19, there were travel restrictions and limited community involvement. Under such circumstances, only one district was picked, which is closer to the state capital (130 kilometres). However, despite its proximity to the capital, Angul is one of the districts with high undernutrition burden. According to the NFHS-5 (National Family Health Survey-4), approximately 44% of WRA women (15–49 years, non-pregnant) and 36% of children (6–59 months) are anaemic (18). In rural Angul, about 22% of children under the age of five are diagnosed with wasting (15).

### Sampling technique

In this study, we have taken women of reproductive age group (WRA, 15–49 years) as the study unit. The households which had at least one member of the WRA group were already enlisted by their respective Gram Panchayat (local self-government), hence sampling frame was available with the respective department of Govt. of Odisha. A simple Random sampling method was used to select the sample. Firstly, 10 Gram Panchayats (GP) were randomly selected from the GP list and those GPs are dispersed over 4 blocks of Angul: Talcher, Kishorenagar, Angul Sadar and Athamalik. Following that, 15 households were selected from each GP randomly from the eligible household list. In total, 150 WRA households were selected and identified for data collection (: sampling technique). The primary data acquired directly from beneficiaries of chosen households (HH) in Odisha’s Angul district.

### Sample size

The sample size required for the beneficiaries was calculated using a population proportion with a specified relative of the main predictor variable, around 55% (19). The reference proportion is available from an adjacent block of the same district which has similar demography and geographical pattern.

The sample size was calculated by using the formula: *n* = 4pq/d^2^, where p (population proportion) = 55% & d (error of margin) = 8.5. Hence, total is 132; considering non-responses & to round up the figure, a total of 150 samples was taken.

### Data source and study participants for qualitative data collection

The government of Odisha has been implementing a special program to promote kitchen garden in rural Odisha for combating undernutrition among vulnerable groups (adolescent girls, pregnant women, lactating mothers & children under 5 years of age) (13). The program includes cash assistance, provision of sapling/seed & capacity building for eligible households for WRA. The inclusion criteria were set accordingly to include the Women of the reproductive age group who have/had a backyard kitchen garden in any of the last three seasons (summer/monsoon/winter). According to the inclusion criteria, households were selected purposively from the Angul district’s two GPs (Angarbandha & Khalari). Two focus group discussions (FGDs) were held with 16 participants from the selected households, 8 for each FGD, to investigate enablers & barriers for sustaining a kitchen garden.

### Measures

#### Outcome variables

The Dietary Diversity Score (DDS) was the only outcome variable used in this study. The DDS was captured as a numerical variable during the data collection, but it was later classified as a binary variable based on the score. DDS was captured using the dietary recall method by a 10-point MDD-W (Minimum Dietary Diversity for Women) scale developed by the FAO and USAID. This pre-validated, easy-to-use, quick, low-cost indicator which counts the food groups consumed by women of reproductive age over the previous 24 h (20). The food groups include (1) Cereals, roots & tubers, (2) Pulses, (3) Nuts & seeds, (4) Milk and milk products, (5) Fish and meat, (6) Eggs, (7) Dark green leafy vegetables, (8) Vitamin A rich vegetables and fruits, (9) Other vegetables, (10) Other fruits. Scoring is done on a 10-point scale, with each food group receiving 1 point for consumption within the last 24 h. Each food group bears one mark for consumption within last 24 h during any meal by the respondent, thereby, the total score was calculated. The dietary diversity was considered as poor/unsatisfactory, in case DDS < 5 and if DDS > =5, categorised as satisfactory dietary quality/diversity. The cut-off marks are pre-decided and validated for pregnant and non-pregnant women (15–49 years) by FAO during this tool’s development, indicating consumption of > = 5 food groups is dietary adequacy (21).

#### Exposure variables

The major independent variables were the presence of a kitchen garden, the HH’s income, the respondent’s occupation, the availability of agricultural land, meat consumption & meal frequency of the participant. The kitchen garden was defined as a small space/land around a homestead, where several species of plants are grown, and their products are primarily intended for family consumption. The variables which showed *p* value <=0.25 during the construction of unadjusted OR, were only considered for further adjustment in the regression model.

### Analytical strategy

#### Quantitative strategy

SPSS 23 was used to conduct the analysis, which included descriptive statistics and multinomial logistic regression. The descriptive analysis examined the sample’s characteristics followed by logistic regression to determine the relationship between the predictor variables and a single outcome variable. The regression was after categorising the outcome variable (DDS) into binary variables based on the DDS score: poor (DDS < 5) and adequate dietary diversity (DDS > = 5). The major independent variables were the presence of a kitchen garden, the HH’s income, the respondent’s occupation, the availability of agricultural land, meat consumption & meal frequency. The variables which showed *p* value <=0.25 during the construction of unadjusted B (OR), were only considered for further adjustment in the regression model.

#### Qualitative strategy

We audio-recorded & transcribed the verbatim and analysed the data. We asked the participants to avoid using names or personally identifying information. Qualitative data were segregated, coded, and analysed through an open coding method manually. Following coding, the data was grouped and checked for emerging patterns to identify themes which led to exploring the enablers & barriers of having a kitchen garden in a rural setting.

## Result

### Quantitative result

A total of 150 samples of the WRA group were taken and their mean age was 31 (Std. Dev. 8.52) (Table 1). The families were largely joint, with 5–6 individuals (mean 5.65) and an average of 0.3 children (under 10 years) per family (Table 1). Most of the beneficiaries interviewed were included (84%) in the SHG (Self Help Group), meaning that at least one member of those HHs used to receive financial assistance from the SHG. A SHG is a community-based group primarily comprised of women from lower socioeconomic backgrounds. They used to borrow from their collective funds in times of urgency or financial constraint, for major life events, or to buy assets. However, most of the families had income below rupees 10,000/− (122$) per month, i.e., meaning that 45% of them were classified as BPL (Below the Poverty Level), with 6% of those being severely poor, meaning that their monthly income was less than rupees 5,000/− (61$) (Table 1). Most of the respondents were homemakers 74.7% (frequency: 112), followed by community cadres 13.3% (frequency: 20) and students 10.7%(frequency: 16) (Table 1). Most of the families owned agricultural land (86%) and almost all of them had their own household toilet/latrine (98%) (Table 1). Furthermore, all the HHs were entitled to receive support from Panchayats for developing KG, but only 67.3% of the HHs had kitchen garden during the previous three seasons (Table 1). To meet their daily vegetable needs, 34 % (34%) of HHs relied mostly on the market, whereas 66% of HHs relied primarily on their kitchen garden (Table 1). Twenty-three per cent of HHs relied only on their agricultural land for their grain needs but most of the HHs relied on PDS (Public Distribution System/Ration: 65%). Ninety-three per cent of the respondents ate at least three times a day, and most of them (95%) were non-vegetarians. The mean DDS was 6.69 with a standard deviation of 7 (Table 1).

**Table 1:** Socio-demographic characteristics of study participants.

Variable	Frequency	Percent	Variable	Frequency	Per cent
Household questions			Food security questions		
Whether the respondent is a SHG member			Is an individual kitchen garden available		
No	24	16.0	No	49	32.7
Yes	126	84.0	Yes	101	67.3
Total	150	100.0	Total	150	100.0
Total family income			The major source of vegetables for the HH		
<5,000	6	4.0	Kitchen garden	99	66.0
>10,000	44	29.3	Market	51	34.0
5,000–10,000	61	40.7	Total	150	100.0
Not responded	39	26.0	The major source of cereals for the HH		
Total	150	100.0	Agricultural land	35	23.3
Occupation of the respondent			Market	17	11.3
Community cadre (ASHA/AWW/ANM/CRP)	20	13.3	PDS*	98	65.3
Homemaker	112	74.7	Total	150	100.0
honey bee maker	1	0.7	Whether consume non-vegetarian food		
Student	16	10.7	No	7	4.7
Ward member	1	0.7	Yes	143	95.3
Total	150	100.0	Total	150	100.0
Availability of agricultural land			Meal frequency		
No	21	14.0	1–2 times	10	6.7
Yes	129	86.0	3 times or more	140	93.3
Total	150	100.0	Total	150	100.0
Availability of HH toilet			*N* = 150	Mean	Std. Deviation
No	3	2.0	Age of the respondent	31.01	8.252
			Total family members	5.65	1.835
Yes	147	98.0	Number of children	0.33	0.564
Total	150	100.0	DDS**	6.69	7
Availability of nearby marketplace					
No	1	0.7			
Yes	149	99.3			
Total	150	100.0			

*PDS, Public Distribution System; **DDS, Dietary Diversity Score.

The multinomial logistic regression model was used after the categorisation of the outcome variable (DDS score) into binary variables based on the DDS score: poor/unsatisfactory (DDS < 5) & satisfactory dietary diversity (DDS > =5). The relevant variables were taken as predictors which include (1) availability of kitchen garden, (2) agricultural land, (3) income, (4) meal frequency, (5) meat consumption and (6) occupation of the respondents. Those variables, which showed *p* value <=0.25 during the construction of unadjusted OR, were only considered for further adjustment in the regression model (Table 2). The result shows absence of backyard kitchen garden is negatively associated with satisfactory dietary diversity (OR: 0.163, *p* = 0.001). In other words, it simply implies that backyard KG is positively associated to satisfactory dietary diversity (Table 2). Further, not having or lack of own Agricultural land also showed a negative association with satisfactory dietary diversity (OR: 0.176, *p* = 0.008) (Table 2). Furthermore, the higher income group demonstrated a positive correlation with satisfactory dietary diversity, i.e., with an increase in income, the DDS also increased. However, statistical significance is absent (Table 2). Three broad occupational categories were used to classify the respondents: students, community workers, and housewives/homemakers. These groups had no discernible impact on the dietary diversity score. Additionally, there was no statistically significant correlation found between the frequency of meals, meat/egg consumption and dietary diversity. Apart from the backyard KG and own agricultural land, the standardised odds ratio (OR) of these SE predictors did not demonstrate a statistically significant correlation. This might explain why having a kitchen garden was strongly associated with increased DDS and not confounded by all these SE predictors.

**Table 2:** Association between kitchen garden & other socio-demographic parameters with DDS.

Parameter estimates				
Dependent variable (binary): satisfactory dietary diversity (DDS > =5)				
Parameter	Unadjusted odds ratio with CI	*p*-value of unadjusted OR	Adjusted odds ratio with CI	*p*-value of adjusted OR
1. Lack or absence of household kitchen garden	0.097 (0.41–0.23)	0.00	0.163 (0.061–0.435)	0.001
2. Lack or absence of own agricultural land	0.103 (0.037–0.28)	0.0	0.176 (0.049–0.630)	0.008
3. Monthly Incomes				
Income= > 10,000	4.44 (1.29–15.24)	0.18	NA**	
Income = 5,000–10000	1.15 (0.47–2.77)	0.755		
Income = <5,000	Ref			
4. Frequency of meal intake				
Meal frequency = 1–2 times	0.182 (0.048–0.68)	0.12	0.538 (0.099–2.91)	0.472
Meal frequency = 3 times or more	Ref		Ref	.
5. Consumption of non-vegetarian food				
Vegetarian	0.780 (0.142–4.2)	0.77	NA**	
Non-vegetarian	Ref			
6. Occupation of the respondent				
Working (community cadre) (ASHA/AWW/ANM/CRP)	0.633 (0.052–7.6)	0.72	0.211 (0.013–3.48)	0.277
Homemaker	0.162 (0.02–1.27)	0.08	0.147 (0.015–1.49)	0.1
Student	Ref		Ref	

**If *p* < =0.25 in unadjusted OR, that variable was considered for further adjustment.

### Qualitative result (thematic analysis)

The FGDs were conducted to identify the factors that enable and hinder rural households’ efforts to grow and manage a kitchen garden in their backyard. Two focus group discussions (FGDs) were held in the Angarbandha and Khalari GPs of the Angul Sadar block on 9.4.22 from 11 AM to 11.45 AM and 14.5.22 from 4 PM to 5 PM, respectively. Every FGD lasted between forty-five and sixty minutes. The criteria for inclusion were used to choose the participants. A total of sixteen women of reproductive age took part in the conversations. The age range of the majority of FGD participants was 25–35 years old. All of them received seed support & cash grants from the local government to develop their kitchen garden. Two FGDs were translated and scripted for thematic analysis. There are 4 major themes identified from arranging the quotes: seasonal variability, initiative of local government, men’s engagement, and challenges (Table 3).

**Table 3:** Thematic tree analysis of responses from FGD.

Themes	Variability in kitchen garden produce throughout the seasons	Initiatives & active support provided by the state & local government			Engagement of male members in developing the KG	Challenges in developing & maintaining the KG
Sub-theme		Hands-on training for beneficiaries	Seed provision to eligible beneficiaries	Cash Grants to eligible beneficiaries		
	Seasonal change	Training & Demonstration	Indigenous seed	Nominal cash	Husbands helped	Water Constraint
	Favourable in winter	Bed rising & mulching	Not hybrid	Cover extra expenses	Coordinated with GP	Lack of land
	Market bought herbs	Organic Fertiliser	Locally grown seeds	Credited as tranche	Heavy work by men	Delay in seed distribution
	Water shortage	Organic Pesticides	Mixing of various plants	Credited to women	Men became accountable	Delay in cash credit
	Drought in summer	Handholding support	Easy to get		Field cleaning	Irregularities in cash grant
		CRP-CM’s role	No extra cost		Manure preparation	
			Good quality			

### Theme 1: variability in kitchen garden produce throughout the seasons

The FGD analysis showed that most participants agreed that the kitchen garden was seasonal and did not produce adequate vegetables to feed the entire family throughout the year, specifically if the family was large. As per them, the kitchen garden was like agricultural crop production. The number of products was higher during winter and less in harsh summer. Almost all the FGD participants agreed that vegetables like potatoes, onion, ginger etc. had to be bought from the market. Only a few families had sufficient financial resources to support the water requirement of the kitchen garden throughout the year, especially during summer.

One of the respondents of FGD: 1 said “Yes, it remains throughout the year but does not yield the same quantity. Summertime sees a decrease, whereas winter and monsoon see a rise.” Another respondent from FGD: 2 said, “It is not possible to grow all varieties of vegetables at the same time, such as potato, tomato, chilli, lemon, and other green vegetables and spinaches together”.

### Theme 2: initiatives and active support provided by the state and local government

The support of local government was identified as one of the important facilitators in developing & maintaining a kitchen garden. This comprised financial assistance, seed distribution, and capacity building for the designated households and eligible beneficiaries. The vulnerable target groups which included adolescent girls, pregnant women & lactating mothers were entitled to receive a packet of indigenous seeds of different plants along with cash assistance. Every participant acknowledged that they received assistance from community volunteers. Additionally, each entitled HH received instruction on how to prepare beds for KG, apply fertiliser, etc.

As per one of the respondents from FGD: 1, “We have received seeds & money. We were given training on how to prepare the bed, do mulching, use pesticides, prepare organic fertilizer etc.” Another respondent from FGD: 2 added, “The provision of seeds is a great help for us. Otherwise, it would not be possible to find quality seeds in the market at a reasonable price.” Many of the participants from both the FGDs said that “receiving all the necessary benefits from Gram Panchayat and the cash grant were helpful to cover expenses for developing our kitchen garden”.

### Theme 3: engagement of male members in developing the kitchen garden

Every participant acknowledged that their husbands had assisted them in growing the kitchen garden. The work done by men was field cleaning, manure pit preparation, “Ranja” preparation, etc. Men’s participation significantly lessened the physical strain placed on women. Being responsible to their female members also aided them. Additionally, according to the respondents, GP’s choice of women as beneficiaries guaranteed their empowerment by granting them nominal cash and directly depositing the money to their bank accounts, which also decreased the likelihood of misappropriation by male members.

One of the respondents from FGD: 1 shared, “Yes, men do help us in preparing this kitchen garden & they used to play an active role in preparing this.” Another respondent from FGD: 2 added, “Men used to take charge of labour-intensive work which needs more physical strength such as field cleaning, manure preparation etc.”

### Theme 4: challenges in developing and maintaining the kitchen garden

The respondents discussed a variety of difficulties they faced while growing their kitchen garden. First, labour-intensive tasks that were impossible for a single person to finish alone. It was particularly challenging for a woman who was pregnant or nursing to continue doing such demanding work. Second, most families did not have a piped water supply, and the kitchen garden used to require a lot of water, which was challenging to provide without one, especially in the summer. Thirdly, the region is surrounded by an industrial belt, and summertime water shortages were typical. Fourth, the respondents also revealed that many of them had not yet received financial assistance and that it used to take a long time to be credited. Lastly, the timing of seed distribution was not appropriate and most of the time they received it after the harvesting period.

One respondent from FGD: 1 said, “I am not able to grow it during the summer because of water constraints.” Another one from FGD: 2 said, “We have received the seed support and training. But there is a problem with crediting the cash.” One of them from FGD: 2 also added, “Seed should be distributed in the proper time just before the season so that harvesting can be done properly”.

## Discussion

This study investigated the relationship between DDS and kitchen garden. A few significant characteristics of rural living standards that had an impact on DDS directly or indirectly were also highlighted in the study. All the respondents were women aged between 15–49 years from the selected HHs. Most of the participants were from lower- or middle-income families, earning less than 10,000 rupees (<$118) a month. The earlier study also showed similar data on the socio-economic status of rural families in India or in Odisha, which used to have several consequences on the nutritional status of women & children (3). The association between poverty and stunting is evident from the significant difference noted in the undernutrition rate among women and children in the lower wealth quintile compared to the higher wealth quintile. In India, the proportion of women with low BMI is 51.5% and stunted children is 59.9% in the lowest wealth index compared with 18.2% of women with low BMI and 25.3% of stunted children in the highest wealth index (NFHS-32006). Regarding occupation, most of the women—roughly 75%—were homemakers, which is precisely in line with the data on women’s employment in rural Odisha (5). This study also tried to clarify the issue of a rural household’s food security. Odisha is a state where agriculture is a predominant profession and this study confirms the same. Eighty-six per cent of the households owned land for farming. But even with their farm, most of the people still got their cereals from PDS (Public Distribution System) which underlines the continued importance of the social protection programmes in rural India. Additionally, this research revealed that 67.3% of homes had backyard kitchen garden, and 66% of those families sourced most of their vegetables from their KG. According to previous studies, a well-developed kitchen garden has the capability of fulfilling the daily dietary requirements of a family by producing nutritionally rich foods such as vegetables, roots, tubers, fruits, legumes, spices etc. (15). Based on previous studies, it was hypothesized that this nutrition-sensitive kitchen garden intervention would be associated with a sustained increase in household dietary diversity leading to better food security, thus indicating an improvement in dietary patterns aimed at reducing undernutrition. Even after standardisation, the KG revealed a statistically significant positive association with good/satisfactory dietary diversity (DDS > =5). This outcome is consistent with earlier research conducted in various contexts, which similarly explains why people who have a kitchen garden have a lower prevalence of inadequate dietary diversity (3, 6). Although no biomarker data were collected on actual micronutrient levels in participants, no conclusions can be reported on the actual nutrient status of participants or household members—a limitation that could be addressed in future studies. The variety of vegetables offered by kitchen garden is likely to contribute to household dietary diversity both directly and indirectly, which is consistent with other researches in similar populations (7, 14–16). Previous research has demonstrated that owning agricultural land is crucial in lowering food insecurity in a variety of contexts (7) and this study’s DDS results were consistent with those of previous studies. Further, another critical predictor ‘income’ had not shown a statistically significant relation with dietary diversity even though earlier research has shown a negative correlation between low income and good dietary outcomes (18, 19). This might be due to the small sample size (only 111 samples) for the correlating income group with dietary diversity (Table 1) i.e. 39 respondents did not answer this question among a total 150 samples. Furthermore, there was no positive correlation observed between DDS and occupation. Based on their responses, the respondents (WRA group) were divided into three occupational categories: homemaker, student, and community worker, indicating that none of them worked in the organised sector and had a fixed source of income. As a result, it was unable to determine how the women’s occupations affected their DDS through accurate comparison. Nonetheless, past research has consistently shown that working women’s greater knowledge leads to improved nutritional outcomes (4, 9).

The qualitative findings are consistent with the quantitative findings regarding the kitchen garden. There were four major themes identified: (1) variability in kitchen garden produce throughout the seasons, (2) initiatives & active support provided by the state & local government, (3) engagement of male members in developing the KG and (4) challenges in developing & maintaining the KG. These themes helped to determine the enablers and barriers associated with KG. Although the kitchen garden is considered as most cost-effective, traditional & sustainable method (3) for ensuring food security, there was hardly any research done previously to examine facilitators or hurdles in the context of Odisha or India. This study clarified important facets of raising a KG. Every participant in the focus group discussion (FGD) concurred that the kitchen garden was seasonal and did not provide enough produce, especially in the summer. However, few families combated it as they were financially stable and had enough resources to support the garden even during the lean season. A study conducted in different developing countries showed how seasonal variation is associated with food production. Second, the support of local government was very significant, which mainly included: capacity building, cash grants and seed support. These things enabled a family to grow a garden easily without financial strain. However, there were certain challenges, such as irregularities in cash grants, delays in seed distribution, and water shortage which demotivated the community members towards this initiative. The men of the HHs actively participated in the growth and upkeep of the kitchen garden, even though its primary beneficiaries were women. Additionally, the financial reward was typically credited to women’s bank accounts, which lessened the likelihood that men would misuse or engage in unethical behavior those are common in rural India.

### Strength and limitation

This study showed the association between different socio-economic factors of rural life and DDS which was largely unexplored in the Odisha context. It also explored the enablers and barriers to developing a KG. This assessment was necessary which may aid in future recommendations & policy formulation. The limitations of this study include direct nutritional status measurement was not taken into consideration. Further, the study did not include the women who did not have kitchen garden in qualitative analysis, which could have given a different perspective on the challenges.

### Areas for further research

Further studies may be carried out measure the diet quality of rural women by using raw food weighment method & biomarker which are more sensitive to measure undernutrition. Research may also be carried out to address the problem of water shortage during summer through innovative agricultural practices so that seasonal food insecurity can be addressed.

## Conclusion

The literature summarised how kitchen garden might enhance Dietary Diversity Score and raise household food security. There are two universal approaches to addressing undernutrition: nutrition-specific or BCC (Behavior Change Communication) intervention and nutrition-sensitive or agricultural intervention. A kitchen garden serves the second approach and increases the accessibility and availability of vegetables at a much lower cost for a family. The support of the local government had played an important role which enabled many of the households to grow a scientifically designed kitchen garden. As the government is focusing on improving the nutritional status of women along with ensuring household food security, it is necessary to put effort into bringing a sustainable solution specifically for rural households & marginalized communities. In this sense, the Kitchen Garden is a cost-effective intervention that can be adopted by the government for nationwide implementation with minimal resources. However, irregularities in cash payment should be addressed for the sustainability of such an initiative. This research will help in generating the evidence which is necessary for policy framing and will help in correcting the loopholes. To summarise, kitchen garden is proving to be a cost-effective approach to increase Dietary Diversity Score. However, for sustainability, support of seed kits and cash assistance is useful.

## Data Availability

The original contributions presented in the study are included in the article/Supplementary material, further inquiries can be directed to the corresponding author.
